# Continuous Non-Invasive Haemodynamic Monitoring During Primary Percutaneous Coronary Intervention

**DOI:** 10.3390/jcm15218307

**Published:** 2026-10-25

**Authors:** Adrian Klatt, Piotr Kasprzyk, Rafał Gałąska, Radosław Targoński, Marek Szołkiewicz, Łukasz Lewicki

**Affiliations:** 1 Department of Cardiology and Interventional Angiology, Kashubian Center for Heart and Vascular Diseases, Pomeranian Hospitals, 84-200 Wejherowo, Poland; 2 Department of Cardiology, Medical University of Gdansk, 80-214 Gdansk, Poland

**Keywords:** ST-elevation myocardial infarction, percutaneous coronary intervention, non-invasive haemodynamic monitoring, volume-clamp method, cardiac index, arterial dP/dt, Acumen IQ, ClearSight

## Abstract

**Background/Objectives:** Haemodynamic assessment during primary percutaneous coronary intervention (PCI) for ST-elevation myocardial infarction (STEMI) is mostly restricted to continuous monitoring of arterial blood pressure. The volume-clamp method provides continuous non-invasive arterial pressure monitoring without the need for vascular access, together with simultaneous estimation of stroke volume, cardiac output and other haemodynamic variables, but its feasibility during primary PCI has not been evaluated. **Methods:** In this prospective observational study, 33 patients with STEMI undergoing primary PCI were monitored continuously (Acumen IQ, HemoSphere) at seven pre-specified timepoints: baseline, guidewire crossing of the culprit lesion, stent implantation, end of procedure, and after 1, 6 and 24 h. Patients with cardiogenic shock, ongoing cardiopulmonary resuscitation or symptom onset more than 24 h before admission were excluded. Cardiac index (CI), stroke volume index (SVI), systemic vascular resistance index (SVRI), pulse rate, dP/dt, mean arterial pressure (MAP) and the Hypotension Prediction Index (HPI) were analysed using linear mixed models with Holm correction within families of related tests. **Results:** Patients were aged 65.4 ± 12.7 years and 25 (76%) were men. The culprit vessel was the left anterior descending artery in 15 patients (45%), and ST-segment resolution exceeding 50% was achieved in 25 patients (76%). The overall time effect was significant for six of seven variables, all except pulse rate. At guidewire crossing, dP/dt rose by 183.7 mmHg/s (95% CI 87–276) in 25 of 29 patients (86%; corrected *p* = 0.008), with a concurrent rise in CI (corrected *p* = 0.042) and no change in pulse rate or MAP. At 24 h, CI, SVI and dP/dt were indistinguishable from baseline, whereas MAP (−13.7 mmHg) and SVRI (−427 dyn·s·cm^−5^·m^2^) were lower. SVI and MAP were unrelated (r = +0.057; *p* = 0.431) across 227 measurements, while SVI and pulse rate were inversely coupled (r = −0.434) in 28 of 33 patients. Increasing age was associated with lower CI and MAP and higher HPI values, but not with the change in arterial dP/dt at guidewire crossing. Patients with TIMI 0 flow had higher SVRI by 510 dyn·s·cm^−5^·m^2^. Baseline pulse rate and its changes were different depending on the culprit vessel. **Conclusions:** Continuous non-invasive haemodynamic monitoring was feasible during primary PCI for STEMI. Given the small sample, the absence of a control group and the lack of validation against a reference method, the findings are exploratory and their clinical value remains to be established.

## 1. Introduction

Acute myocardial infarction with ST-segment elevation (STEMI) is a condition in which total ischaemic time is the principal determinant of infarct size and outcome. The primary objective of treatment is restoration of blood flow through the occluded vessel by percutaneous coronary intervention (PCI) [1,2]. In the catheterisation laboratory information on the patient’s condition is limited mostly to invasive arterial pressure, electrocardiography and bedside echocardiography. Advanced invasive haemodynamic monitoring is not performed routinely. As the transradial approach is standard in primary PCI [1,2], additional invasive monitoring would require a separate vascular access, which is not always technically straightforward, may delay the procedure when time is critical, and carries a risk of additional complications. Detailed information on the patient’s circulatory status was, at that time, unavailable.

The volume-clamp method [3] requires no vascular access. Applied through a finger cuff, it reconstructs a continuous arterial waveform and derives stroke volume, cardiac output, systemic vascular resistance, mean arterial pressure, arterial dP/dt, defined as the maximal rate of rise in arterial pressure, and the Hypotension Prediction Index [4]. It adds information the arterial line cannot provide, and continues uninterrupted into the intensive care period after the sheath is removed.

The technique has been validated against thermodilution by Bubenek-Turconi et al. before and after preload manipulation [5], by Wang et al. in open-chest cardiac surgery [6], by Yahagi et al. and Stolte et al. during structural cardiac interventions [7,8]. No consensus exists on interchangeability for absolute cardiac output values [9,10,11,12]. However, these studies indicate the method might be clinically useful in interventional settings, particularly for analysing haemodynamic trends [13,14]. Nevertheless, a recent meta-analysis has reported limited accuracy and precision of continuous non-invasive blood pressure devices [15]. Likewise, a meta-analysis of minimally invasive pulse wave analysis devices concluded that neither their accuracy nor their trending ability was sufficient for interchangeability with reference techniques [16].

The utility of the volume-clamp method has not yet been evaluated during primary PCI for STEMI. Mechanical reperfusion is a discrete event whose circulatory consequences unfold over seconds to minutes, a timescale that intermittent measurement cannot resolve. The aim of this investigation was to characterise the haemodynamic profile of STEMI patients undergoing primary PCI using continuous non-invasive monitoring. The method, by design, was supposed to be used starting at baseline, through the stages of intervention and the following twenty-four hours, to determine whether this identifies changes in cardiac output and arterial dP/dt not accessible through pressure monitoring or post-procedural echocardiography. The study was designed to assess feasibility and to generate physiological hypotheses. It was not designed to validate cardiac output estimates against a reference method [13] or to evaluate clinical utility.

## 2. Methods

### 2.1. Study Design and Setting

This was a prospective observational study conducted at the Kashubian Center for Heart and Vascular Diseases, aiming to characterise the haemodynamic profile of patients undergoing percutaneous coronary intervention for ST-segment elevation myocardial infarction. The study was approved by the hospital administration and by the Bioethics Committee of the Regional Medical Chamber in Gdańsk (no. KB37/26) in accordance with the principles of Good Clinical Practice. It was designed as an exploratory, hypothesis-generating pilot investigation. The sample size was determined pragmatically by the number of consecutive eligible patients enrolled during the study period rather than by a pre-specified target. No subgroup comparisons or specific research hypotheses were pre-specified at the design stage. All subgroup analyses reported in this manuscript are exploratory.

Patients were excluded if there was ongoing cardiopulmonary resuscitation, limb dysmorphia precluding cuff placement, cardiogenic shock, or chest pain onset more than 24 h before admission. Patients had to be conscious, haemodynamically and respiratorily stable at baseline. Patients with valvular heart disease were not excluded. A detailed assessment was not possible before primary PCI because time was crucial. However, post-procedural echocardiography showed no severe valvular disease in any patient. Patients receiving inotropic or vasopressor support before the procedure were excluded under the cardiogenic shock criterion, whereas those who required such treatment during the procedure or in the intensive cardiac care unit remained in the study. Written informed consent was obtained in the catheterisation laboratory, jointly with consent for coronary angiography, by the principal investigator, who was present at every procedure and was not the operator. This took place while the nursing team prepared the patient and the operator prepared for the procedure, so reperfusion was not delayed. The cuff was applied afterwards, before the sterile field was prepared. After clinical stabilisation in the intensive cardiac care unit, the study was discussed again with each patient. No patient declined participation or later withdrew consent.

Patients were enrolled between August 2026 and September 2026. A total of 35 consecutive patients with a clinical diagnosis of STEMI were enrolled, and 33 were included in the analysis after angiographic confirmation.

### 2.2. Non-Invasive Haemodynamic Monitoring

Monitoring was performed using the HemoSphere Advanced Monitoring Platform (instrument software version 3.1) with the Acumen IQ finger cuff (ClearSight technology; Edwards Lifesciences, Irvine, CA, USA). The system applies the volume-clamp method of Peňáz [3] combined with the Physiocal calibration algorithm [17] to reconstruct a continuous finger arterial pressure waveform. The finger waveform is subsequently transformed into a brachial arterial pressure waveform [17]. Stroke volume is estimated from the systolic area of the reconstructed signal using a pulse-contour algorithm incorporating a three-element Windkessel model individualised for age, sex, height and weight.

**Figure 1:**
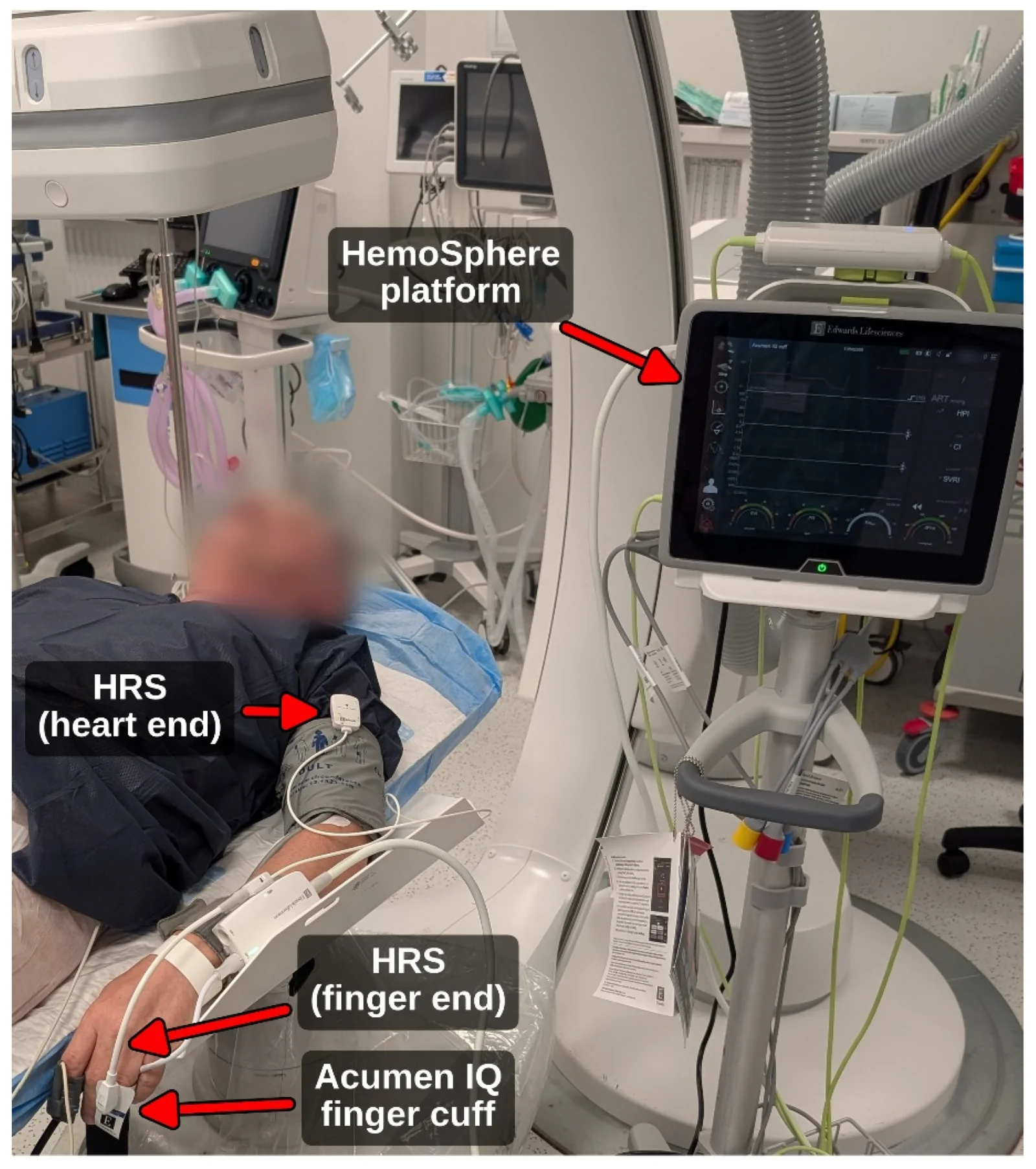
Monitoring setup in the catheterisation laboratory. HRS, Heart Reference Sensor. Source: authors’ own collection.

The Acumen IQ cuff was most commonly placed on the middle phalanx of the second or fourth finger of the hand contralateral to the vascular access site (the left hand in 32 patients and the right hand in 1 patient) to avoid interference with the arterial waveform signal from the access site. Arterial access for PCI was obtained via the right radial artery in 31 patients, the left radial artery in 1 and the right femoral artery in 1. The patient with femoral access had the cuff placed on the left hand. A reference heart-level sensor was positioned at the mid-axillary line and connected to the HemoSphere base unit (Figure 1). The cuff was applied before vascular access was obtained; monitoring continued throughout the procedure and for the following 24 h in the intensive cardiac care unit. Signal quality was verified by visual inspection of the arterial waveform. Upper-limb pulses were not assessed separately before monitoring. Signal adequacy was judged from the arterial waveform and the signal quality indicator of the HemoSphere platform, which was satisfactory in all patients once the signal had stabilised. The monitored finger was rotated every 4–6 h by nursing staff, per manufacturer recommendation, to prevent pressure injury or pain.

Key procedural events were timestamped against the HemoSphere device clock, recorded either on a separate procedural log or directly on the device. Every case was monitored by the same investigator, who was present throughout the procedure. All haemodynamic data were recorded automatically by the monitor and exported as a Microsoft Excel file (Microsoft 365; Microsoft Corporation, Redmond, WA, USA) using the native HemoSphere export function. Values were extracted into the analysis spreadsheet as the mean of the shortest available averaging interval provided by the device software (20 s).

### 2.3. Haemodynamic Variables

Seven parameters were analysed: (MAP), (PR), (SVI), (CI), (SVRI), (dP/dt), and (HPI).

MAP is the time-weighted average of the arterial pressure waveform within each cardiac cycle (area under the curve divided by cycle duration), updated with every beat. PR is derived from the interval between peaks of the arterial pressure waveform (the pulse-wave equivalent of the R–R interval). SVI is estimated by the pulse-contour method from the systolic area of the pressure waveform, individualised by a three-element Windkessel model incorporating age, sex, height and weight [4], and normalised to body surface area (DuBois formula). CI is calculated as SVI × PR/1000—an algorithmically derived quantity rather than an independently measured one. SVRI is calculated as 79.9 × (MAP − CVP)/CI, where central venous pressure (CVP) is assumed at a fixed value of 5 mmHg (the manufacturer’s default setting), since the non-invasive device does not measure it directly. SVRI is therefore a second quantity algebraically dependent on MAP and CI. dP/dt is the maximum slope of the systolic upstroke of the arterial pressure waveform. It reflects the rate of rise in the reconstructed arterial pressure waveform and also depends on arterial compliance and loading conditions. HPI is a proprietary, machine-learning-based algorithm (Edwards Lifesciences) trained on features extracted from the arterial pressure waveform, returning a value from 0 to 100 that reflects the probability of a hypotensive event (MAP < 65 mmHg) within the following 15 min. The exact architecture of the algorithm is not publicly disclosed by the manufacturer. HPI has not been validated as a predictor of hypotension in acute STEMI, and its predictive performance was not assessed in this study; it is reported here as a device output only.

All values updated automatically every 20 s.

### 2.4. Timepoints

Seven pre-specified timepoints were defined (Table 1).

For timepoints T1–T4, given the high dynamics of change during the procedure, the value entered into the analysis was the single shortest available extracted average (20 s). Where the procedural event did not coincide with a complete 20 s averaging cycle, the value from the next complete cycle following the event was used. For timepoints T5–T7, a 60 s average was used, calculated across three consecutive extracted points (the target point and the one immediately preceding and following it).

TIMI flow in the infarct-related artery was additionally assessed retrospectively from the separate procedural log and from archived angiograms at the first contrast injection after guidewire crossing. Flow was categorised as absent (TIMI 0) or present (TIMI > 0).

### 2.5. Statistical Analysis

#### 2.5.1. Principal Model: Linear Mixed Model

Models of the form variable ~ time (categorical, seven levels) + random patient intercept were fitted by maximum likelihood, and the time effect assessed by likelihood ratio testing against a model without time (6 degrees of freedom). Contrasts are reported as fixed-effect coefficients with 95% confidence intervals. Three considerations motivated this choice. Missing data are structural. Eight of 33 patients (24%) lacked at least one value—four had no dP/dt or HPI recording, two had no stent timepoint, two had no 24 h data—so listwise deletion, as required by repeated-measures analysis of variance, would have discarded a quarter of the cohort together with all their remaining measurements. The mixed model uses every available observation. Sphericity was violated for all seven variables (Mauchly test *p* = 0.020 to <0.001), which in a repeated-measures analysis of variance would require Greenhouse–Geisser correction with an attendant loss of power. The mixed model makes no sphericity assumption. Between-patient variability was substantial (intraclass correlation 0.29–0.72), requiring a random intercept.

#### 2.5.2. Verification

Principal results were reproduced under restricted maximum likelihood and under an alternative optimiser (Powell in place of BFGS), with identical conclusions. Repeated-measures analysis of variance with Greenhouse–Geisser correction, run as a sensitivity analysis on complete cases, gave concordant directions but lower power: SVRI (*p* = 0.069) and HPI (*p* = 0.062) lost significance, reflecting both the smaller effective sample and the correction itself.

#### 2.5.3. Non-Parametric Tests for Transitions

Changes between consecutive timepoints were assessed with the Wilcoxon signed-rank test supplemented by the sign test. These make no distributional assumption and allow reporting of the proportion of patients responding in the expected direction, which is more informative than the mean alone. Confidence intervals for the change at guidewire crossing were obtained by bootstrap resampling with 10,000 replicates.

#### 2.5.4. Within-Patient Correlations

A correlation pooled across all observations would be confounded by between-individual differences (non-independence of observations). Repeated-measures correlation [18] estimates a common slope after accounting for the patient effect, with adjusted degrees of freedom (df = N − number of patients − 1). It was applied only to non-tautological pairs—combinations of stroke volume index, pulse rate, mean arterial pressure and dP/dt—since cardiac index and systemic vascular resistance index are computed by the device algorithm from these quantities rather than measured independently. Volume-clamp pressure measurement remains accurate during atrial fibrillation [14], but stroke volume estimation in this rhythm has not been validated. As sensitivity analyses, repeated-measures correlations were therefore repeated after excluding the four patients with atrial fibrillation. They were also repeated after removing the cohort mean at each timepoint to account for shared procedural trends.

#### 2.5.5. Subgroup Analyses

All subgroup and covariate analyses were exploratory. Factors were selected on clinical grounds as likely to influence haemodynamics (age, pre-procedural TIMI flow, culprit vessel) rather than on the basis of the data, and their results are hypothesis-generating. Models of the form variable ~ time + group and variable ~ time × group were compared. Testing the former against a model without a group detects a difference in level (1 degree of freedom); testing the latter against the former detects a difference in trajectory shape (6 degrees of freedom). The distinction matters interpretatively: a difference in level without interaction indicates that groups start from different values but respond to the procedure alike. Separate tests at each timepoint were deliberately avoided, as this would multiply comparisons and ignore the repeated-measures structure. Continuous variables were compared with the Mann–Whitney test and categorical variables with Fisher’s exact test.

#### 2.5.6. Correction for Multiple Testing

The Holm–Bonferroni method was applied within each family of related comparisons, defined by the structure of the analysis (one family per type of comparison): 7 omnibus tests of the time effect, 42 contrasts against a given reference timepoint, 42 transitions between adjacent timepoints, 7 per subgroup analysis (main effects and interactions corrected separately), 7 per continuous covariate, and 6 within-patient correlations. The procedure is uniformly more powerful than the Bonferroni correction while retaining strong control of the family-wise error rate. Contrasts against the baseline and against the end of the procedure were treated as two separate families; where a comparison belonged to more than one family, it is reported for the family in which it was tested.

Age was not tested as separate correlations with each derived feature—under that approach no result survives correction—but as a covariate in the mixed model, which amounts to seven tests rather than fifty-six while using all timepoints.

#### 2.5.7. Software

Analyses were performed in Python 3.12 (Python Software Foundation, Wilmington, DE, USA) using statsmodels 0.15.0 (mixed models), pingouin 0.6.1 (repeated-measures correlation and analysis of variance, Mauchly test), and scipy 1.17.1 (non-parametric tests, bootstrap). Two-sided tests were used throughout, with significance assessed at 0.05 on Holm-corrected *p* values.

## 3. Results

### 3.1. Study Population and Data Completeness

Thirty-three of the thirty-five enrolled patients were included (age 65.4 ± 12.7 years, range 29–83; 25 men, 76%). Two enrolled patients were excluded because, despite chest pain and electrocardiographic changes, coronary angiography showed no culprit lesion in the epicardial coronary arteries. Diagnosis of STEMI was not confirmed. The left anterior descending artery was the culprit vessel in 15 (45%) and TIMI 0 flow was present before intervention in 20 (61%). Single-vessel disease was present in 20 (61%) and multivessel disease in 13 (39%), including 6 (18%) with a chronic total occlusion of a non-culprit artery. PCI was limited to the culprit lesion in all patients, and no staged revascularisation was performed during the 24 h monitoring period. Final TIMI 3 flow was achieved in 31 (94%); the remaining two, both with TIMI 0 flow before intervention, had final TIMI 2 flow. ST-segment resolution exceeding 50% was achieved in 25 (76%) and 27 (82%) presented within 6 h of symptom onset. Atrial fibrillation was present in 4 (12%). Ejection fraction was 43.9 ± 9.0% after PCI and 51.9 ± 10.1% at one month (*n* = 20). Baseline characteristics are given in Table 2.

Of a maximum of 1617 measurements, 1533 (94.8%) were available. In two patients the culprit vessel was recanalised without stenting on the basis of the angiographic result, so T3 does not exist and was treated as structurally absent rather than missing. In four patients dP/dt and HPI were not recorded owing to irreversible Acumen IQ sensor malfunction; in two, 24 h data were lost through mechanical damage to the finger cuff. Numbers contributing to each timepoint are shown in the figures. No measurement was excluded on the basis of signal quality. Apart from the baseline, recorded after signal stabilisation, shown by the device’s signal quality indicator, all values were taken at fixed positions relative to the timestamped procedural events (Section 2.4).

Electrocardiographic reperfusion was defined as ST-segment resolution on the post-procedural tracing relative to admission, dichotomised at 50%. Ejection fraction was measured by transthoracic echocardiography (Vivid E95, GE HealthCare, Chicago, IL, USA; left lateral decubitus, Simpson biplane) within one hour of the procedure and at one month.

### 3.2. Peri-Procedural Haemodynamic Course

The overall time effect was significant for six of seven variables, indicating a difference between at least two timepoints: MAP (χ^2^ = 26.5, *p* = 0.001), dP/dt (χ^2^ = 24.5, *p* = 0.002), SVRI (χ^2^ = 20.1, *p* = 0.013), SVI (χ^2^ = 19.8, *p* = 0.013), CI (χ^2^ = 19.7, *p* = 0.013) and HPI (χ^2^ = 16.9, *p* = 0.019), all Holm-corrected across the seven variables. Pulse rate showed no significant time effect (χ^2^ = 6.2, *p* = 0.403). CI is calculated from SVI and pulse rate, and SVRI from MAP and CI, while HPI is derived from the arterial waveform by a proprietary algorithm of the manufacturer. Of the four independently derived variables, three changed significantly: MAP, SVI and dP/dt. Mean values at each timepoint are given in Table 3. Between-patient variability accounted for a substantial share of total variance (intraclass correlation 0.29–0.72).

**Figure 2:**
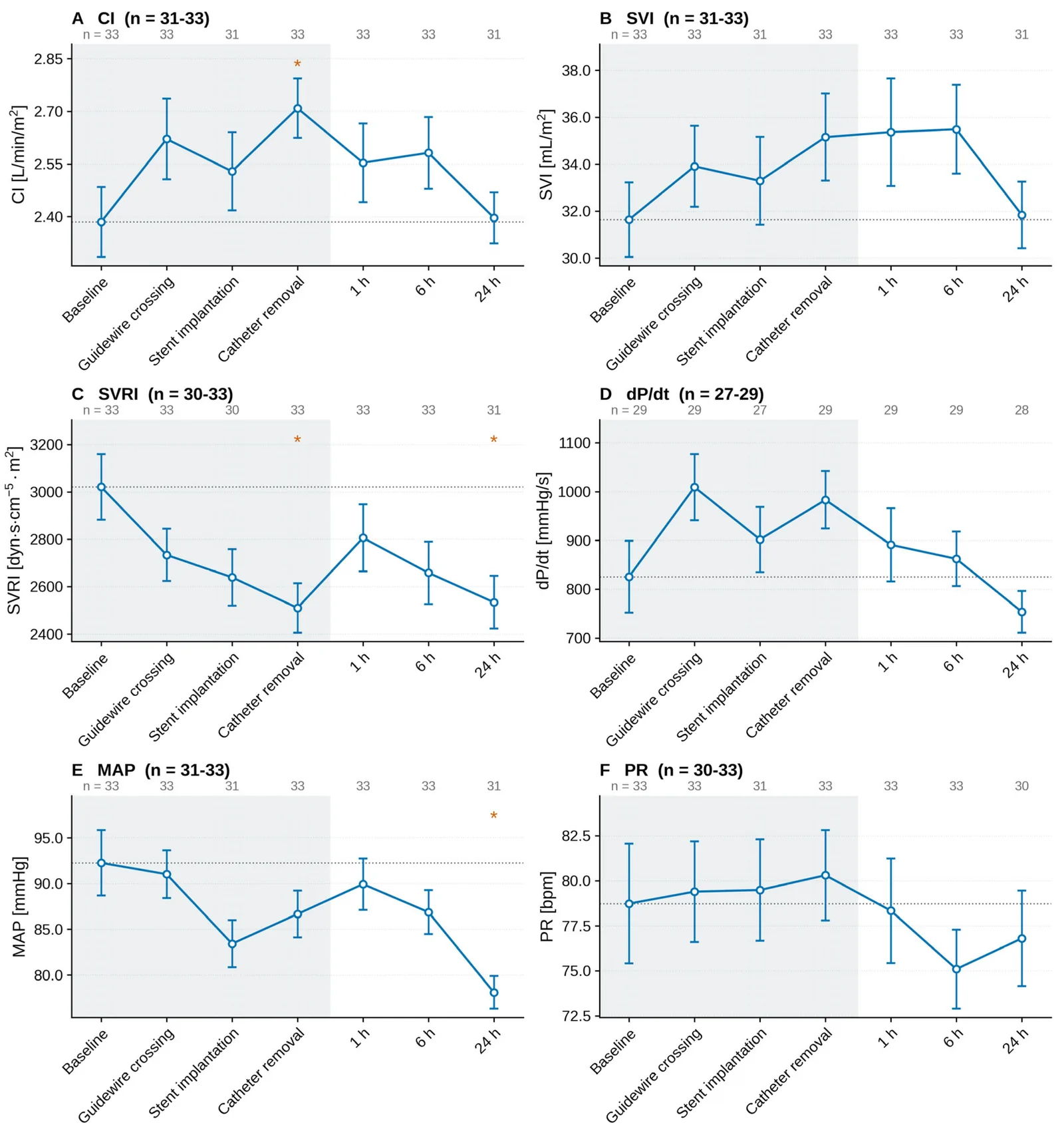
Peri-procedural trajectories of six haemodynamic variables. Values are mean with standard error of the mean. The shaded band marks the procedural phase and the dotted line the baseline value. Numbers above each panel give the patients contributing a measurement at that timepoint. * Holm-corrected *p* < 0.05, for the contrast against the baseline within a family of 42 contrasts from linear mixed models. T1 baseline, T2 guidewire crossing, T3 stent implantation, T4 catheter removal, T5 1 h, T6 6 h, T7 24 h. CI, cardiac index; SVI, stroke volume index; SVRI, systemic vascular resistance index; dP/dt, rate of rise in arterial pressure; MAP, mean arterial pressure; PR, pulse rate.

Of the 42 contrasts against the baseline, four remained significant after Holm correction (Figure 2): MAP at 24 h (−13.74 mmHg, 95% CI −19.81 to −7.66; *p* = 0.0004), SVRI at end of procedure (−511 dyn·s·cm^−5^·m^2^, −759 to −264; *p* = 0.002), CI at end of procedure (+0.32 L/min/m^2^, +0.14 to +0.51; *p* = 0.019) and SVRI at 24 h (−427 dyn·s·cm^−5^·m^2^, −679 to −175; *p* = 0.035).

### 3.3. Changes at Guidewire Crossing

**Figure 3:**
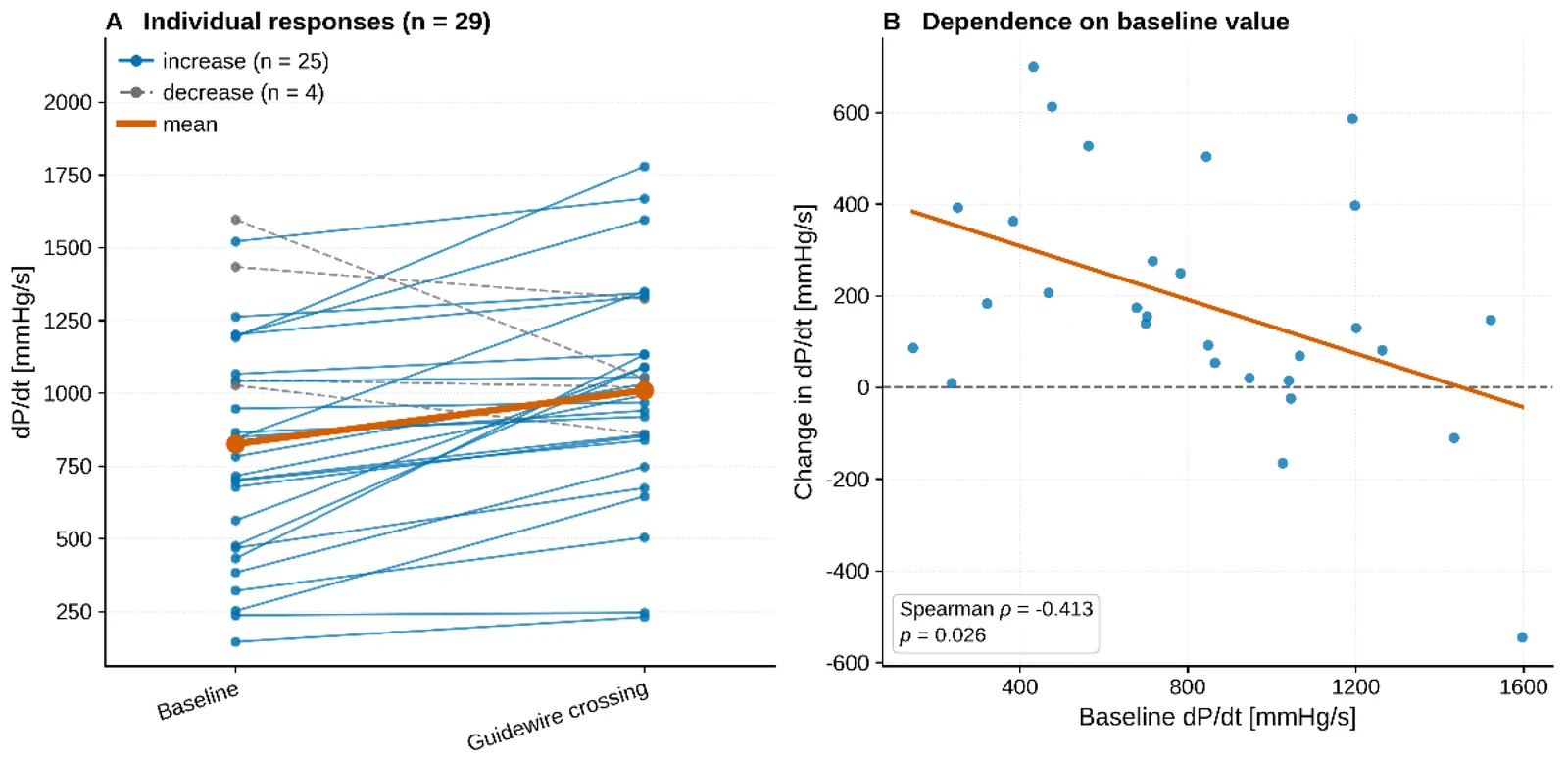
Arterial dP/dt at guidewire crossing. (**A**) Individual dP/dt values at baseline and at guidewire crossing; solid lines an increase, dashed lines a decrease, the heavy line the group mean. (**B**) Magnitude of the response against the baseline value. The confidence interval is a percentile bootstrap interval from 10,000 replicates.

Of 42 transitions between adjacent timepoints, two remained significant after Holm correction, both at guidewire crossing. dP/dt increased by 183.7 mmHg/s (bootstrap 95% CI 87 to 276; median +147) in 25 of 29 patients (86%; Figure 3); Wilcoxon *p* = 0.0002, corrected *p* = 0.008. CI increased concurrently by 0.24 L/min/m^2^, rising in 22 of 33 and remaining unchanged in 8 (corrected *p* = 0.042). Neither pulse rate nor MAP changed at this timepoint.

Among the 20 patients with TIMI 0 flow at baseline, after guidewire crossing, at the first contrast injection TIMI > 0 flow was present in 14 of them. dP/dt data were unavailable in 4 of these 20 patients. Among the remaining 16, dP/dt increased in 10 of 11 patients with restored flow (mean +199 mmHg/s, bootstrap 95% CI 78 to 337) and in 3 of 5 without it (+8 mmHg/s, −284 to 260). In patients with TIMI 1–2 flow before the procedure, dP/dt increased in 12 of 13 (+238 mmHg/s, 117 to 364). Changes in CI, SVI and SVRI at guidewire crossing were similar regardless of flow restoration. Given the small group sizes, these comparisons are descriptive.

The magnitude of the dP/dt change was inversely related to baseline dP/dt (ρ = −0.413, *p* = 0.026): below the median an increase occurred in 14 of 14 patients (+291 mmHg/s), against 11 of 15 (+84 mmHg/s) above it.

### 3.4. Status at 24 Hours

**Figure 4:**
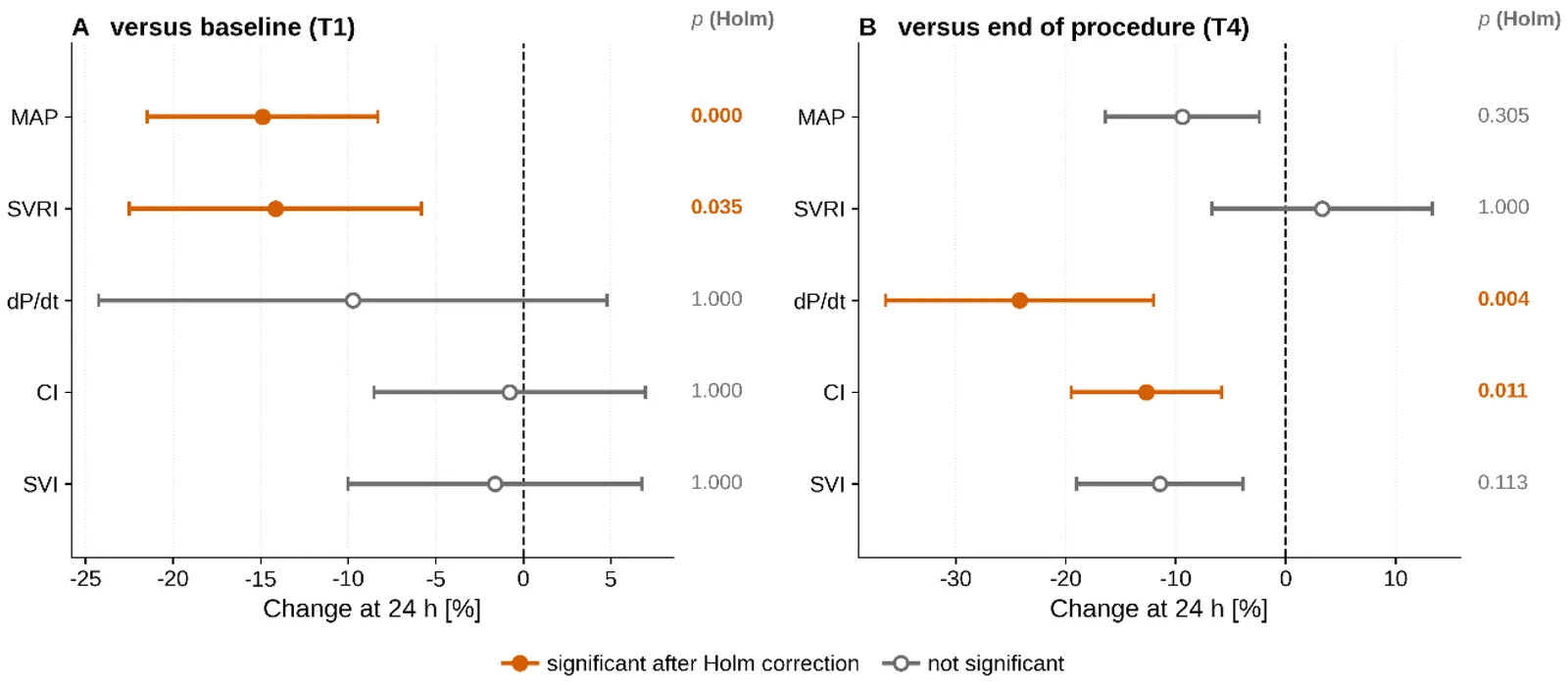
Haemodynamic status at 24 h assessed against two reference points: (**A**) the pre-procedural baseline and (**B**) the end of the procedure. Points are percentage changes with 95% confidence intervals from linear mixed models. Filled symbols denote contrasts significant after Holm correction within each family of 42 contrasts; open symbols denote non-significant contrasts.

Relative to baseline, only the vascular variables differed at 24 h (Figure 4): MAP was lower by 13.74 mmHg and SVRI by 427 dyn·s·cm^−5^·m^2^ (both corrected, as above), whereas CI (*p* = 0.846), SVI (*p* = 0.708) and dP/dt (*p* = 0.189) did not differ from pre-procedural values. Relative to the end of the procedure, dP/dt was lower by 237.7 mmHg/s (95% CI −358 to −118; corrected *p* = 0.004) and CI by 0.34 L/min/m^2^ (−0.53 to −0.16; *p* = 0.011); SVI (−4.02 mL/m^2^; *p* = 0.114) and MAP (−8.13 mmHg; *p* = 0.305) did not survive correction within this family.

### 3.5. Effect of Age

**Figure 5:**
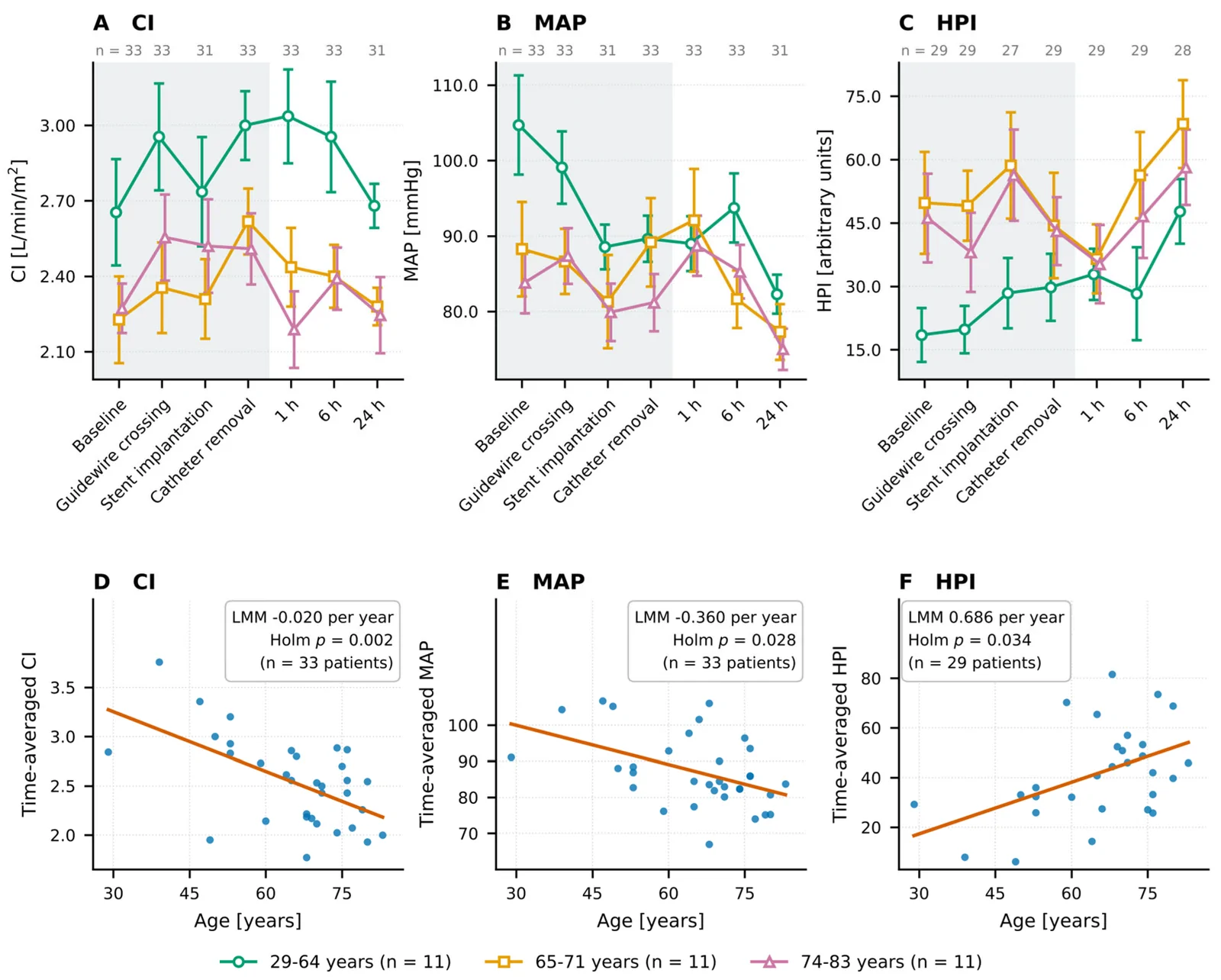
Effect of age. (**A**–**C**) Trajectories by age tertile, mean with standard error of the mean. (**D**–**F**) Per-patient values averaged across timepoints, with an ordinary least-squares regression line. The coefficient quoted in each panel is the age main effect from the linear mixed model fitted to all available observations, not the slope of the plotted line. Tertiles were formed by rank, giving 11 patients each.

Age was entered as a covariate in the mixed models. Three main effects survived Holm correction (Figure 5): CI (−0.020 L/min/m^2^ per year, 95% CI −0.030 to −0.010; *p* = 0.002), MAP (−0.360 mmHg per year, −0.595 to −0.125; *p* = 0.028) and HPI (+0.686 points per year, +0.220 to +1.152; *p* = 0.034)—per decade, a CI lower by 0.20 L/min/m^2^, MAP lower by 3.6 mmHg and HPI higher by 6.9 points.

Age × time interactions were significant for SVI (*p* = 0.033), pulse rate (*p* = 0.038) and MAP (*p* = 0.029), indicating that age modified trajectory shape as well as level. dP/dt showed no relationship with age, either in level (−0.67 per year, *p* = 0.861) or in trajectory shape (*p* = 0.882).

### 3.6. Within-Patient Associations

**Figure 6:**
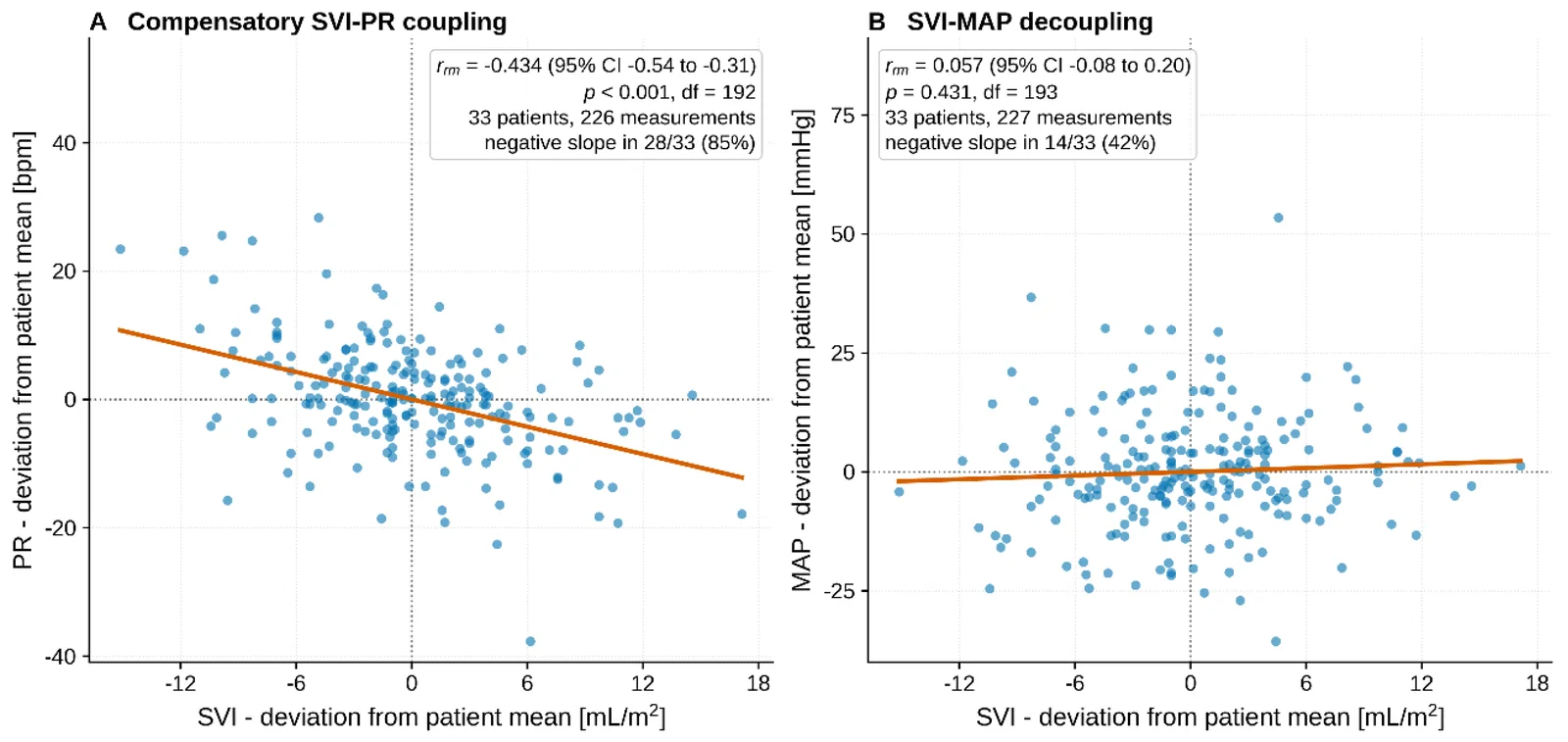
Within-patient associations for (**A**) stroke volume index and pulse rate and (**B**) stroke volume index and mean arterial pressure. Values are centred within each patient, so each point is one measurement expressed as a deviation from that patient’s own mean. r_rm, repeated-measures correlation coefficient.

Repeated-measures correlations were calculated for the four independently measured variables; all pairs were significant after Holm correction with one exception (Table 4). The strongest association, between dP/dt and MAP, should be interpreted with caution. Both are derived from the same arterial pressure waveform, so part of this correlation reflects their shared dependence on blood pressure rather than an independent physiological relationship. The inverse SVI–pulse rate relationship (r = −0.434, 95% CI −0.54 to −0.31) was present in 28 of 33 patients (85%; sign test *p* = 0.00007). SVI and MAP were not associated within patients (r = +0.057, −0.08 to +0.20; *p* = 0.431); the confidence interval narrowly bracketing zero across 227 measurements (Figure 6). Both findings were unchanged after adjustment for timepoint (SVI–pulse rate r = −0.458, *p* < 0.001; SVI–MAP r = +0.027, *p* = 0.713) and after exclusion of the four patients with atrial fibrillation (r = −0.388, *p* < 0.001, and r = +0.062, *p* = 0.421, respectively).

### 3.7. Pre-Procedural TIMI Flow

Patients with TIMI 0 flow (*n* = 20) were compared with TIMI 1–2 (*n* = 13). The groups did not differ in age (*p* = 0.768), sex (*p* = 1.000), culprit vessel (*p* = 1.000), ST-segment resolution (*p* = 1.000), time from symptom onset (*p* = 0.364), atrial fibrillation (*p* = 1.000) or ejection fraction (*p* = 0.490).

**Figure 7:**
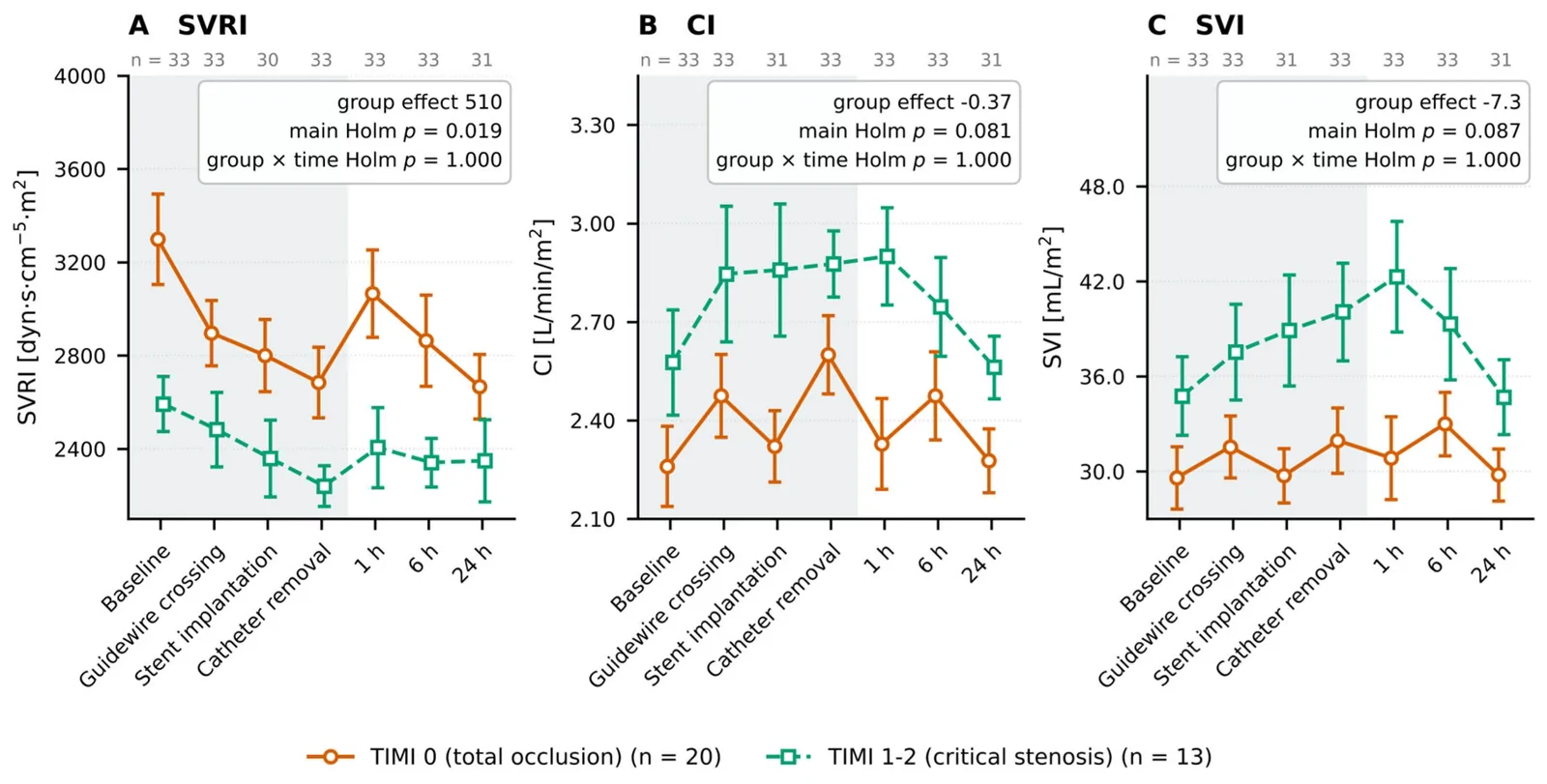
Haemodynamic course according to pre-procedural TIMI flow in the infarct-related artery. Values are mean with standard error of the mean. The group effect is the main effect from the linear mixed model; the interaction term tests whether trajectory shape differs between groups.

SVRI was higher by 510 dyn·s·cm^−5^·m^2^ in the TIMI 0 group (95% CI 199 to 820; corrected *p* = 0.019). CI was lower by 0.37 L/min/m^2^ (−0.66 to −0.09; *p* = 0.081) and SVI by 7.29 mL/m^2^ (−13.04 to −1.54; *p* = 0.087), neither surviving correction. All group × time interactions were non-significant (*p* ≥ 0.26), indicating a difference in level rather than trajectory shape (Figure 7).

### 3.8. Culprit Vessel Location

**Figure 8:**
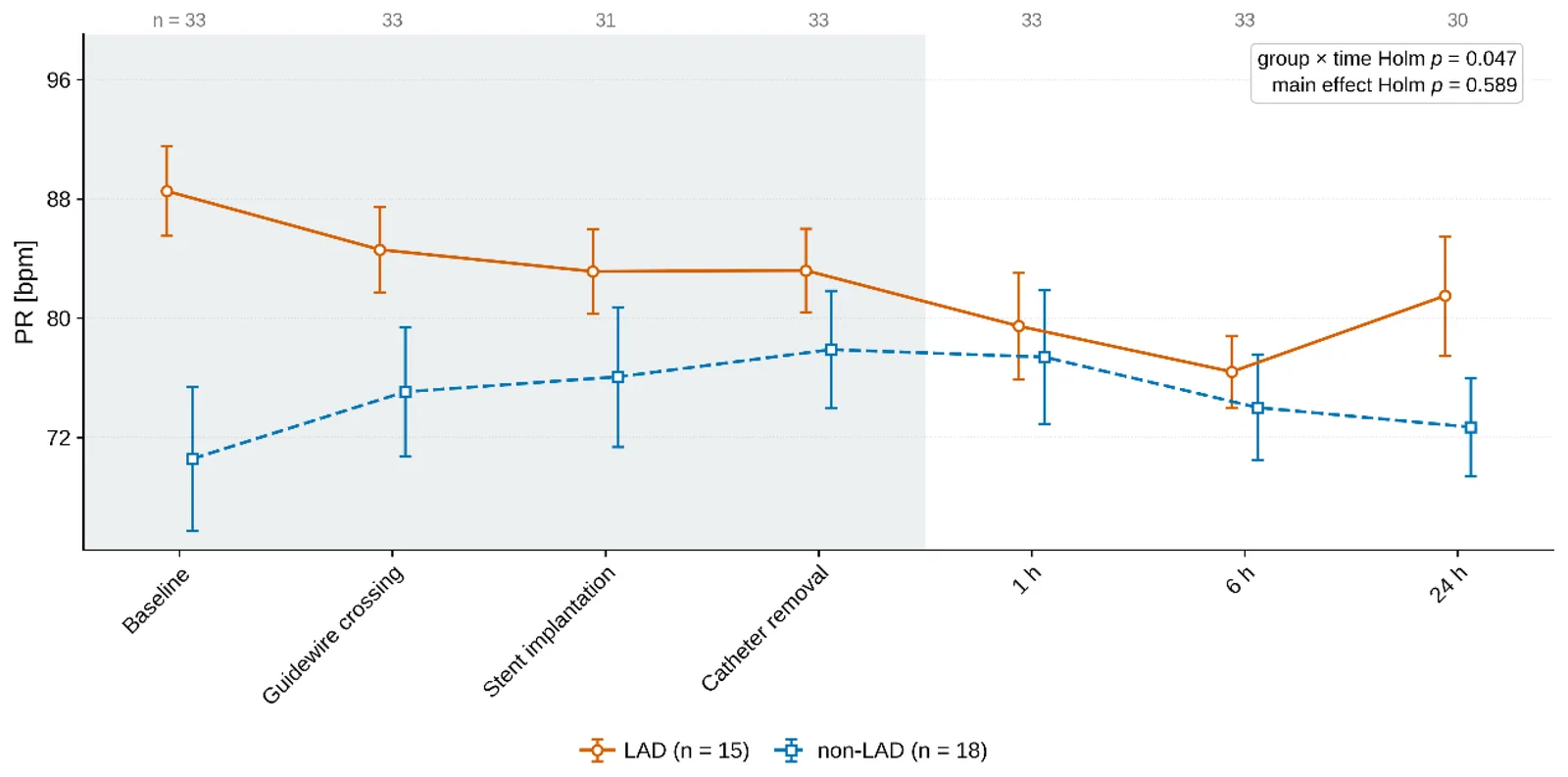
Pulse-rate trajectory according to culprit vessel location. Values are mean with standard error of the mean. LAD, left anterior descending artery.

LAD (*n* = 15) and non-LAD (*n* = 18) infarctions were compared. The group × time interaction for pulse rate was significant (corrected *p* = 0.047; Figure 8); no main effect and no other interaction reached significance. Baseline pulse rate was higher in the LAD group (88.5 ± 11.6 vs. 70.6 ± 20.5 bpm, *p* = 0.003) and the intra-procedural change ran in opposite directions, falling by 5.3 bpm in LAD and rising by 7.3 bpm in non-LAD (*p* = 0.018); by the end of the procedure the groups no longer differed (*p* = 0.247). Ejection fraction was lower in the LAD group (39.9 ± 9.2 vs. 47.2 ± 7.4%, *p* = 0.034).

## 4. Discussion

### 4.1. Principal Findings

Continuous non-invasive monitoring identified an immediate rise in arterial dP/dt and cardiac index at guidewire crossing, loss of that improvement by 24 h, and a dissociation between arterial pressure and stroke volume within individual patients. Given the exploratory design, all interpretations below are provisional.

### 4.2. Internal Consistency of the Measurements

The volume-clamp method has not been validated against an invasive reference in STEMI [9,19], so the internal coherence of the recordings warrants comment. Two features support the internal consistency of the recordings.

Pulse rate—the one variable with no expected relation to the procedural sequence—showed no time effect whatever, which non-specific device drift would be unlikely to spare. And the exported values were algebraically consistent throughout, with cardiac index matching the product of stroke volume index and pulse rate within 0.1 L/min/m^2^ in 93% of records; the remaining differences reflect rounding of the exported values and separate averaging of each variable over the sampling interval.

Some caution remains warranted. Cardiac index and systemic vascular resistance index are computed by the algorithm from stroke volume, pulse rate and pressure, so significant time effects in these two are partly inherited from their inputs rather than independent confirmation; only four of the seven variables are independently derived.

### 4.3. Arterial dP/dt at Guidewire Crossing

The most consistent change at guidewire crossing was a rise in arterial dP/dt. This variable is the maximal slope of the systolic upstroke of the reconstructed finger arterial pressure waveform, not left ventricular dP/dtmax. It depends on arterial compliance, vascular tone and loading conditions as well as on ventricular function. Systemic vascular resistance index fell over the same interval, so part of the increase is likely to reflect altered loading rather than a change in intrinsic contractility. Cardiac index rose concurrently; as pulse rate did not change, the increase was carried by stroke volume, and the fall in resistance would facilitate ejection irrespective of any change in contractile function.

The analysis of TIMI flow after guidewire crossing addresses the origin of this change only in part. Among patients with an occluded artery, the rise in arterial dP/dt was largely restricted to those in whom flow was restored (10 of 11 patients, +199 mmHg/s), whereas in those without restored flow dP/dt was essentially unchanged (+8 mmHg/s). This pattern is consistent with a reperfusion-related component. However, a comparable rise occurred in patients whose artery was already patent before the procedure (12 of 13 patients, +238 mmHg/s), and changes in CI, SVI and SVRI did not depend on flow restoration. Procedural factors common to this stage, such as contrast injection, catheter manipulation and concomitant medication, are therefore likely to contribute, and the two cannot be separated in the present data. The groups were small, particularly the six patients without flow restoration, so this comparison is descriptive and hypothesis-generating.

Importantly, the observed restoration of flow cannot be attributed to guidewire crossing alone. Unfractionated heparin was administered to all patients after the baseline measurement (T1) and before guidewire crossing, and heparin-related thrombolysis or spontaneous recanalisation may have restored flow in some patients before the wire reached the lesion. TIMI flow was assessed only at the first contrast injection after guidewire crossing, so it cannot be determined whether flow returned because of heparin, the wire passage, or both.

If a reperfusion-related component is present, it may reflect the metabolic consequences of restored perfusion. Ischaemia depletes ATP and lowers intracellular pH, impairing cross-bridge cycling, sarcoplasmic reticulum calcium reuptake and myofilament calcium sensitivity. Reperfusion restores oxidative phosphorylation and washes out accumulated metabolites, with recovery of ATP and pH within minutes, which is compatible with the timescale observed here. Such recovery would be distinct from myocardial stunning, which by definition persists after reperfusion and resolves over hours to days. Angiographic restoration of epicardial flow does not, however, guarantee myocardial reperfusion. Reperfusion injury and microvascular obstruction may limit tissue-level perfusion despite a patent artery [20]. Tissue-level reperfusion was not assessed. ST-segment resolution, the only available marker, was recorded after the procedure.

The magnitude of the rise was inversely related to its baseline value, with an increase in 14 of 14 patients below the median, averaging +291 mmHg/s, against 11 of 15 averaging +84 above it. This gradient should be interpreted with caution, because a change analysed against its own baseline is prone to regression to the mean, which the present design cannot exclude.

### 4.4. Decline by 24 Hours

Relative to the end of the procedure, dP/dt and cardiac index had declined. This observation cannot be attributed to a single mechanism. Twenty-nine of 33 patients (88%) received a beta-blocker within 24 h, which reduces arterial dP/dt and cardiac output directly, and no untreated comparison group was available. Pain, anxiety and the procedure itself raise sympathetic tone, and resolution of this acute adrenergic state may also contribute, but in the presence of near-universal beta-blockade the two effects cannot be separated. Ischaemia–reperfusion injury, including microvascular obstruction, would produce a similar time course [20,21] and cannot be excluded, but no markers of microvascular or inflammatory injury were collected, and relative to the pre-procedural baseline all three variables had returned to their starting values rather than falling below them. The Hypotension Prediction Index was highest at 24 h. At this timepoint it was closely and inversely related to MAP across patients (Spearman ρ = −0.80), with the highest values in patients whose MAP was close to or below 65 mmHg. Its rise therefore largely mirrors the fall in MAP. Whether it carries information beyond MAP, or predicts hypotension in STEMI, cannot be determined from these data, as the algorithm has not been validated in this setting.

### 4.5. Arterial Pressure and Stroke Volume

Stroke volume index and mean arterial pressure were unrelated within patients across 227 measurements. This should not be read as physiological independence—stroke volume is one determinant of pressure, alongside resistance and rate. A possible explanation is compensation. The inverse association between stroke volume index and pulse rate, present in 28 of 33 patients, is consistent with a fall in stroke volume being offset by a rise in rate. This would leave pressure unchanged while output declines. The reverse direction—reduced diastolic filling at higher rates—is equally compatible with these data. Both findings were unchanged after adjustment for timepoint and after exclusion of patients with atrial fibrillation. They may, however, still reflect treatment effects, and correlation alone does not establish a mechanism. A similarly weak within-patient relationship between arterial pressure and cardiac index has been reported in patients undergoing major abdominal surgery, in whom blood pressure was a poor surrogate for cardiac index [22].

The clinical implication is that a stable mean arterial pressure does not guarantee a stable stroke volume. Because pressure is defended by compensatory mechanisms of finite reserve, it may remain unremarkable until that reserve is exhausted, at which point it falls abruptly, so that pressure may be a late rather than an early indicator; this hypothesis cannot be tested in the present data. This reserve is plausibly narrower where chronotropic response is limited, as in beta-blockade, sinus node disease or atrial fibrillation, though the present cohort was too small to examine this. We note only that pressure, the single haemodynamic variable routinely monitored in the catheterisation laboratory, cannot substitute for flow.

### 4.6. Interpretation of the Age Effect

Cardiac index and mean arterial pressure fell, and the Hypotension Prediction Index rose, with advancing age—per decade, a cardiac index lower by 0.20 L/min/m^2^, a pressure lower by 3.6 mmHg and a hypotension index higher by 6.9 points. Of these, only MAP is measured directly; CI is derived by an algorithm that uses age as an input, and HPI has not been validated as a predictor of hypotension in acute STEMI, so its higher values do not establish a higher risk of hypotension.

If these associations are genuine, a biological reading is straightforward: reduced myocardial and vascular reserve, diminished β-adrenergic responsiveness and impaired diastolic function would all lower output and limit compensation, implying that an infarct of equivalent size may be less well tolerated in an older patient.

A methodological explanation is equally possible. Age is itself an input to the Windkessel model, so the association between age and cardiac index may be partly algorithmic rather than biological. Pulse-contour estimation rests on a Windkessel model calibrated on population rather than individual data, so age-related arterial stiffening or poorer digital perfusion could bias the estimate independently of any true difference [23]. Against this, dP/dt—derived from the same waveform—showed no age relationship, whereas stiffening would be expected to steepen the systolic upstroke and raise it. Resolution would require an invasive comparator.

### 4.7. Pre-Procedural TIMI Flow and Vascular Resistance

Systemic vascular resistance index was higher by 510 dyn·s·cm^−5^·m^2^ throughout the observation period in patients presenting with TIMI 0 flow, with no difference in trajectory shape—a shift in level rather than in response. Complete cessation of antegrade flow implies a larger ischaemic territory with stronger sympathetic activation and reflex vasoconstriction, whereas preserved, though slowed, inflow may afford partial protection. The groups were balanced for every clinical variable examined, including ejection fraction, so this difference was apparent to continuous monitoring but not to conventional assessment. The comparison rests on 20 versus 13 patients and was not pre-specified.

### 4.8. Pulse Rate and Culprit Vessel

Pulse rate followed opposite intra-procedural courses, falling by 5.3 bpm in LAD and rising by 7.3 bpm in non-LAD infarction. The baseline difference agrees with the known autonomic pattern of acute infarction, described from heart rate and blood pressure [24] and from heart rate variability [25]: vagal predominance in inferior infarction, attributed to the Bezold–Jarisch reflex, and sympathetic predominance in anterior infarction. This imbalance is attenuated by revascularisation [25], and the groups no longer differed by the end of the procedure. Withdrawal of vagal tone may also unmask underlying sympathetic activation [24], which would contribute to the rise in rate in non-LAD infarction. Reperfusion of the inferior wall can transiently intensify the reflex, with abrupt bradycardia and hypotension [26], but such brief episodes would not be captured at the predefined timepoints. A further candidate explanation is transient sinus node ischaemia: the sinus node artery arises from the right coronary artery in roughly 60% of individuals and the circumflex in the remainder, so non-LAD occlusion may compromise nodal perfusion. This would account for the slow baseline rhythm in that group, with acceleration as perfusion is restored. In LAD infarction the nodal artery is spared, and the baseline rate of 88 bpm represents an unopposed adrenergic response. Increased vagal tone in inferior infarction would produce the same pattern without nodal ischaemia; the two cannot be distinguished, as nodal artery origin was not recorded.

## 5. Limitations

This was an exploratory pilot study without pre-specified hypotheses or power calculation, and subgroup analyses rest on small numbers, requiring confirmation. The volume-clamp method was not validated against an invasive reference in this population, and cardiac index and systemic vascular resistance index are algorithm-derived rather than independently measured [19]. SVRI was calculated with a fixed central venous pressure of 5 mmHg (the manufacturer’s default). Actual central venous pressure varies between patients and over time. This may affect both absolute SVRI values and their changes over time. Pronounced peripheral vasoconstriction and arrhythmia—both more common in the sickest patients—degrade pulse-contour reconstruction [23]. Upper-limb arterial stenosis, including subclavian stenosis, was not specifically excluded before monitoring. The single-use sensor is susceptible to mechanical damage during prolonged monitoring. There was no control group, and no simultaneous invasive reference measurements were obtained. These would have required a separate vascular access, which, as noted in the Introduction, may prolong the procedure. The study was therefore designed to use non-invasive measurements only. TIMI flow after guidewire crossing was assessed retrospectively, and the subgroup without flow restoration was small (*n* = 6, five with dP/dt recordings). Twenty-nine of 33 patients (88%) received a beta-blocker within 24 h, precluding separation of its effect from the natural course. Peri-procedural medications administered during PCI, including sedatives, vasoactive and analgesic agents, as well as contrast injections, may also have influenced the recorded haemodynamic values. Their timing relative to the measurement windows was not recorded, so periods following these interventions could not be excluded from the analysis. Time from symptom onset was recorded only as a binary variable; echocardiographic follow-up was limited, and the study was conducted at a single centre by one investigator.

## 6. Conclusions

Continuous non-invasive haemodynamic monitoring appears feasible during primary PCI for STEMI and records haemodynamic trajectories in the acute phase that are not apparent from arterial pressure alone. These trajectories were not related to clinical outcomes in this study. Given the small sample, the absence of a control group and the lack of validation against a reference method, these findings are exploratory. Confirmation of its clinical value will require larger, invasively validated studies.

## Figures and Tables

**Table 1:** Pre-specified monitoring timepoints.

#	Timepoint	Operational Definition
T1	Baseline	After cuff signal stabilisation and sheath insertion, before drug administration
T2	Guidewire crossing	Guidewire crossed the culprit lesion
T3	Post-stenting	After balloon deflation post-stenting (last stent, if >1)
T4	End of procedure	Immediately after catheter withdrawal
T5	1 h	1 h after end of procedure
T6	6 h	6 h after end of procedure
T7	24 h	24 h after end of procedure

**Table 2:** Baseline characteristics of the cohort (*n* = 33).

Variable	Value
Age, years	65.4 ± 12.7 (29–83)
Male sex	25 (76%)
Height, cm	170.0 ± 8.5
Body weight, kg	80.9 ± 20.4
Body surface area, m^2^	1.91 ± 0.25
LAD as culprit vessel	15 (45%)
Pre-procedural TIMI 0 flow	20 (61%)
TIMI >0 after guidewire crossing among TIMI 0 patients	14 (70%) (*n* = 20)
ST resolution > 50%	25 (76%)
Symptom onset < 6 h	27 (82%)
Atrial fibrillation	4 (12%)
LVEF after PCI, %	43.9 ± 9.0
LVEF at 1 month, %	51.9 ± 10.1 (*n* = 20)

Values are mean ± SD (range) or *n* (%). LAD—left anterior descending artery; LVEF—left ventricular ejection fraction.

**Table 3:** Haemodynamic variables at each timepoint (mean ± SD).

Variable	T1	T2	T3	T4	T5	T6	T7
CI, L/min/m^2^	2.38 ± 0.57	2.62 ± 0.66	2.53 ± 0.62	2.71 ± 0.49	2.55 ± 0.64	2.58 ± 0.59	2.40 ± 0.40
SVI, mL/m^2^	31.6 ± 9.1	33.9 ± 9.9	33.3 ± 10.4	35.2 ± 10.6	35.4 ± 13.1	35.5 ± 10.8	31.8 ± 7.9
SVRI, dyn·s·cm^−5^·m^2^	3021 ± 797	2734 ± 634	2639 ± 655	2510 ± 599	2806 ± 817	2658 ± 757	2534 ± 619
PR, bpm	78.7 ± 19.1	79.4 ± 16.0	79.5 ± 15.7	80.3 ± 14.4	78.3 ± 16.7	75.1 ± 12.6	76.8 ± 14.5
dP/dt, mmHg/s	826 ± 396	1009 ± 364	902 ± 348	983 ± 316	891 ± 405	862 ± 302	754 ± 226
MAP, mmHg	92.3 ± 20.6	91.0 ± 14.9	83.4 ± 14.3	86.7 ± 14.7	89.9 ± 16.2	86.9 ± 13.8	78.1 ± 9.9
HPI	37.8 ± 32.7	35.2 ± 26.7	46.7 ± 33.3	38.9 ± 29.3	34.9 ± 23.9	43.3 ± 33.3	58.1 ± 27.9

T1 baseline, T2 guidewire crossing, T3 stent implantation, T4 end of procedure, T5 1 h, T6 6 h, T7 24 h. *n* = 30–33 for CI, SVI, SVRI, PR and MAP; *n* = 27–29 for dP/dt and HPI. Means were calculated from individual measurements. SVRI was calculated by the device for each measurement, so applying the SVRI formula to the table means does not exactly reproduce the mean SVRI. Differences reported in the text are contrasts from linear mixed models and may differ from differences between table means when data are missing at a timepoint, as at 24 h.

**Table 4:** Within-patient (repeated-measures) correlations.

Pair	r (95% CI)	df	*p*
dP/dt–MAP	+0.553 (0.44 to 0.65)	170	<0.0001
SVI–PR	−0.434 (−0.54 to −0.31)	192	<0.0001
dP/dt–SVI	+0.275 (0.13 to 0.41)	170	0.001
dP/dt–PR	+0.266 (0.12 to 0.40)	169	0.001
PR–MAP	+0.230 (0.09 to 0.36)	192	0.003
SVI–MAP	+0.057 (−0.08 to 0.20)	193	0.431

*p* values are Holm-corrected across the six pairs.

## Data Availability

The data presented in this study are available on request from the corresponding author, and are not publicly available. Data contain information that could compromise participant privacy.

## Abbreviations

The following abbreviations are used in this manuscript:

**Abbreviation**	**Definition**
CI	cardiac index
dP/dt	maximum rate of arterial pressure rise
HPI	Hypotension Prediction Index
LAD	left anterior descending artery
LVEF	left ventricular ejection fraction
MAP	mean arterial pressure
PCI	percutaneous coronary intervention
PR	pulse rate
STEMI	ST-segment elevation myocardial infarction
SVI	stroke volume index
SVRI	systemic vascular resistance index
TIMI	Thrombolysis in Myocardial Infarction flow grade
